# Randomized controlled trial of favipiravir, hydroxychloroquine, and standard care in patients with mild/moderate COVID-19 disease

**DOI:** 10.1038/s41598-022-08794-w

**Published:** 2022-03-23

**Authors:** Manaf AlQahtani, Nitya Kumar, Dhuha Aljawder, Abdulkarim Abdulrahman, Mohammed Wael Mohamed, Fatema Alnashaba, Mohammed Abu Fayyad, Faisal Alshaikh, Fatima Alsahaf, Sawsan Saeed, Amal Almahroos, Zainab Abdulrahim, Sameer Otoom, Stephen L. Atkin

**Affiliations:** 1 National Task Force for Combating the Corona Virus (COVID-19), Riffa, Bahrain; 2 Bahrain Defence Force Hospital, Riffa, Bahrain; 3 Royal College of Surgeons in Ireland, Riffa, Bahrain; 4 Salmaniya Medical Complex, Manama, Bahrain; 5 Sheikh Mohammed Bin Khalifa Cardiac Centre, Riffa, Bahrain; 6 King Hamad University Hospital, Muharraq, Kingdom of Bahrain

## Abstract

Favipiravir has antiviral activity against influenza, West Nile virus, and yellow fever virus and against flaviviruses. The objective of this pilot study was to compare three arms: favipiravir; hydroxychloroquine; standard care (no specific SARS-CoV-2 treatment) only, in symptomatic patients infected by SARS-CoV-2 in an open-labelled randomized clinical trial. The trial was registered with Bahrain National Taskforce for Combatting COVID-19 on the 7th of May 2020 (registration code: NCT04387760). 150 symptomatic patients with COVID-19 disease were randomized into one of three arms: favipiravir, hydroxychloroquine, or standard care only. The primary outcome was the clinical scale at the end of study follow up (day 14 or on discharge/death) based on a points scale. The secondary outcomes were viral clearance, biochemical parameter changes and mortality at 30-days. Baseline characteristics did not differ between groups. The proportion of patients who achieved a clinical scale < 2 did not differ between groups. The favipiravir-treated and hydroxychloroquine-treated group showed increased viral clearance (OR, 95%CI 2.38, 0.83–6.78, OR, 95%CI 2.15, 0.78–5.92, respectively) compared to standard care, but this was not significant. The biochemical profile did not differ between groups, except for the platelet count (*P* < 0.03) and uric acid (*P* < 0.004) that were higher with favipiravir-treatment. Primary or secondary outcome measures did not differ between favipiravir, hydroxychloroquine, and standard therapy for mild to moderate COVID-19 disease; therefore, whilst favipiravir therapy appeared safe with a trend to increased viral clearance, there was no superior therapeutic utility.

Clinical trials registration. NCT04387760. Registration date: 07/05/2020.

## Introduction

Coronavirus disease 2019 (COVID-19) is caused by the severe acute respiratory syndrome coronavirus 2 (SARS-CoV-2) and has developed into a pandemic with serious global public health and economic sequelae. As of May 30th, 2021, more than 170,695,962 million cases have been confirmed worldwide, leading to over 3,550,234 deaths^1^. Several vaccines have been shown to prevent or ameliorate COVID-19 disease, and several are currently developing^2^. However, for those that establish COVID-19 disease, there is a need for effective therapeutic agents to prevent the progressive deterioration that may be seen. There have been several reports of medications, such as remdesivir, with antiviral properties that have shown efficacy with shorter time-to-recovery against SARS-CoV-2^3^. It has been suggested that corticosteroids should be used cautiously unless there is evidence of refractory septic shock, acute respiratory distress syndrome (ARDS), or another compelling indication for their Use^4^. Hydroxychloroquine was thought to show great promise at the time that this study was initiated^5^ but, in a large observational study, showed no differences in rates of intubation or death^6^, and a randomized trial in 150 patients showed mild beneficial effects^7^. A subsequent report indicated that postexposure therapy with hydroxychloroquine did not prevent COVID-19 disease^8^. Azithromycin, often used in conjunction with hydroxychloroquine, has also been used in COVID-19 disease but whether it has beneficial effects is unclear^9^. Additional therapeutic options, such as convalescent plasma, appear safe in clinical practice^10^ and may be of benefit, though the timing of its use and who may benefit the most is still unclear^11^.

Favipiravir is an antiviral drug developed in Japan that is a pyrazinecarboxamide derivative with activity against viruses causing influenza, West Nile fever, yellow fever, foot, and mouth disease, and against flaviviruses (i.e., arenaviruses, bunyaviruses, and alphaviruses)^12^. Its mode of action is through inhibition of viral RNA-dependent RNA polymerase^13^. In February 2020, favipiravir was used to treat COVID-19 disease in China and was declared effective as a treatment^14^. A report comparing favipiravir with lopinavir /ritonavir suggested that favipiravir was superior for preventing COVID-19 disease progression and viral clearance^15^. The objective of this pilot study was to compare three arms: favipiravir; hydroxychloroquine; standard care (no specific SARS-CoV-2 treatment) only, in symptomatic patients infected by SARS-CoV-2 in an open-labelled randomized clinical trial. The difference between groups would allow an effect size to be determined for a definitive clinical trial.

## Methods

This was a prospective, randomized, controlled open-labeled pilot study involving patients with symptomatic COVID-19 disease confirmed by RT-PCR testing^16^. All patients gave written informed consent. This study was approved by the National COVID-19 Research Committee and the Bahrain Defence Force Hospital Ethics committee and was conducted in accordance with the Declaration of Helsinki and local regulations. The trial was conducted using the approval of the National Research and Ethics committee.

### Participants

Patients were recruited from two medical centres in Bahrain. The study recruitment was from August 2020 to March 2021; when all needed participants were recruited.

### Inclusion criteria

Inclusion criteria were: (1) signed informed consent; (2) aged at least 21 years; (3) COVID-19 diagnosis based on polymerase chain reaction (PCR) testing; (4) symptomatic with any COVID-19 symptoms requiring admission to hospital; (5) Hypoxia as defined by an oxygen saturation (SpO2) of more than or equal to 93% on air, or a PaO_2_ to FiO_2_ ratio of more than 300 on enrolment.

### Exclusion criteria

Exclusion criteria were the following: (1) Severe COVID-19 disease: defined as presence of SpO_2_ less than 93% on room air or a PaO_2_ to FiO_2_ ratio of 300 or lower at time of enrolment; (2) Patients on ventilatory support; (3) Cardiac dysfunction that would preclude treatment with hydroxychloroquine; (4) Renal dysfunction (estimated glomerular filtration rate less than 30 ml/min); (5) Hepatic dysfunction; (6) Gout or a history of gout; (7) Pregnant or breastfeeding women; (8) Patients with a known allergy to an intervention medication; (9) Patients with glucose-6-phosphatase deficiency; (10) Readmission due to Covid-19 disease; (11) Participants in any other COVID-19 disease trial; (12) Patients on immunosuppressants, HIV patients, cancer patients who received chemotherapy within the past 6 months, or who were on chronic oral steroids.

### Randomization

**Figure 1:**
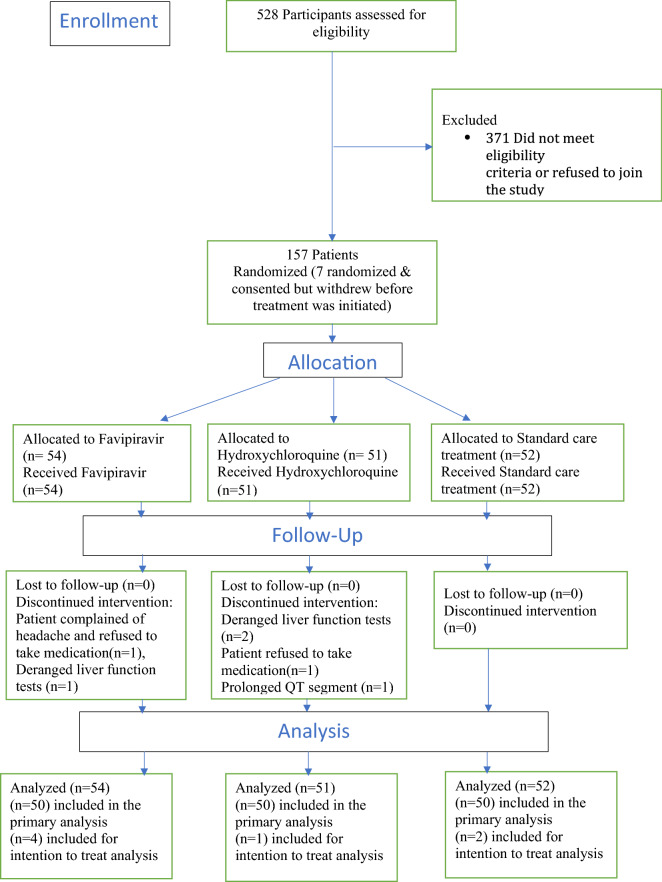
Flow chart of patient participation in the clinical trial.

Following informed consent and screening, the patients were block randomised (in blocks of 4) by computer-generated random numbering to the favipiravir (1600 mg twice daily orally day one only, subsequently 600 mg BID PO days 2 to 10), hydroxychloroquine therapy (400 mg twice daily orally day one only, subsequently 200 mg twice daily orally from day 2 to day10) or standard treatment arms (Fig. 1). The physicians on site would call the research coordinator to randomise the patients using the computer-generated random numbering, once deemed eligible and have given informed consent. The participants are then randomised to one of three arms of the study. Patients and clinicians were not blinded to the treatment given.

### Standard supportive treatment

The standard supportive treatment included control of fever (paracetamol) and possible therapy antibacterial medication.

### Trial procedure

Following informed consent, inclusion and exclusion criteria were determined, and patient demographic data were collected (Table 1). Medical history was ascertained, including current smoking status (including pack-year history), diabetes mellitus, heart disease, chronic lung disease, chronic liver disease, chronic kidney disease, asthma, HIV, tuberculosis. For potential recruits, the severity of the COVID-19 presentation was captured, including shortness of breath and oxygen therapy. Anthropometric measurements were collected, including BMI, blood pressure, temperature, respiratory rate, heart rate, oxygen saturation on room air, and oxygen requirement. An ECG was done on all participants. Laboratory measures conducted included: Full Blood Count (Hb, WBC, Neutrophil count, Neutrophil percent (%), Lymphocyte count, Lymphocyte percent (%), Platelets, Blood Group, Biochemical profile: Na, K, Urea, Creatinine, liver function tests C-reactive protein, Ferritin, D-dimer, lactate, and uric acid (Table 2). A pregnancy test was done for all females randomized to favipiravir.

**Table 1:** Baseline characteristics of the participants across the treatment groups.

Characteristics	Treatment Group			
	Favipiravir	Hydroxychloroquine	Standard Care	*P*-value*
N	54	51	52	
Age [median (IQR)]	44.5 (33.0, 50.0)	40.0 (34.0, 56.0)	48.5 (35.5, 57.0)	0.17
BMI, Kg/m^2^ [median (IQR)]	29.6 (25.6, 31.6)	27.9 (24.2, 32.1)	28.7 (26.4, 32.5)	0.56
Male [n(%)]	23 (43%)	25 (50%)	27 (52%)	0.60
Smoker [n(%)]	0 (0%)	2 (4%)	3 (6%)	0.21
Diabetes [n(%)]	12 (22%)	9 (18%)	20 (38%)	0.040
Heart Disease [n(%)]	1 (2%)	0 (0%)	1 (2%)	0.61
Chronic Lung Disease or Asthma [n(%)]	6 (11%)	2 (4%)	4 (8%)	0.38
Shortness of Breath [n(%)]	14 (26%)	15 (29%)	18 (35%)	0.62
Oxygen therapy administered [n(%)]	0 (0%)	0 (0%)	1 (2%)	0.36
Ventilation Required [n(%)]	0 (0%)	0 (0%)	1 (2%)	0.36
Temperature, °C [median (IQR)]	36.8 (36.6, 37.0)	36.8 (36.8, 37.1)	36.8 (36.6, 37.0)	0.49
Systolic BP, mmHg [median (IQR)]	122.0 (110.0, 136.0)	120.0 (114.0, 133.0)	122.5 (112.0, 134.5)	0.87
Diastolic BP, mmHg [median (IQR)]	75.0 (70.0, 83.0)	72.0 (65.0, 79.0)	72.0 (64.0, 79.0)	0.22
Respiratory rate, breaths/min, [median (IQR)]	18.0 (18.0, 20.0)	18.0 (18.0, 20.0)	18.0 (18.0, 20.0)	0.83
Heart rate, beats/min, [median (IQR)]	80.0 (73.0, 89.0)	84.0 (77.0, 90.0)	83.0 (73.5, 89.5)	0.51
Oxygen Saturation, mmHg [median (IQR)]	98.0 (96.0, 99.0)	98.0 (96.0, 98.0)	97.0 (96.0, 98.0)	0.17

*P-values are based on Kruskal–Wallis test.

**Table 2:** Blood Count and Biochemical Profile of the participants at baseline [median (IQR)].

Parameter	Treatment Group			
	Favipiravir	Hydroxychloroquine	Standard Care	*P*-value*
N	54	51	52	
Hemoglobin mg/dl	13.3 (12.2, 14.4)	14.0 (12.1, 15.4)	13.5 (12.3, 14.6)	0.14
White Blood Cells	4.7 (3.9, 6.3)	4.9 (4.3, 6.2)	4.7 (3.5, 6.8)	0.99
Neutrophil Count	3.0 (2.0, 4.0)	3.0 (2.0, 4.0)	3.0 (2.0, 4.0)	0.61
Neutrophil Precent	60.3 (52.1, 66.4)	59.7 (51.6, 66.7)	62.2 (51.5, 71.9)	0.69
Lymphocyte Count	1.3 (0.8, 1.6)	1.3 (0.9, 1.7)	1.1 (0.8, 1.5)	0.39
Lymphocyte Percent	29.6 (22.9, 36.7)	29.4 (24.9, 38.4)	26.7 (21.0, 37.1)	0.47
Platelets	224.5 (174.0, 303.0)	188.0 (146.0, 226.0)	192.0 (149.0, 234.0)	0.012
Sodium mmol/l	138.0 (137.0, 140.0)	138.0 (136.0, 141.0)	139.0 (136.0, 140.5)	0.73
Potassium mmol/l	4.3 (4.0, 4.6)	4.3 (3.9, 4.6)	4.5 (4.1, 4.8)	0.090
Urea mmol/l	4.2 (3.3, 5.0)	3.9 (3.0, 4.5)	4.7 (3.6, 6.0)	0.002
Creatinine umol/l	61.5 (51.0, 73.0)	60.0 (50.0, 71.0)	61.5 (54.0, 71.5)	0.76
Lactate dehydrogenase mmol/l	217.0 (174.0, 271.0)	211.0 (178.0, 262.0)	214.0 (182.0, 311.5)	0.76
C-Reactive Protein mmol/l	16.8 (4.0, 32.1)	14.9 (4.2, 32.5)	17.4 (5.3, 58.4)	0.42
Uric Acid mmol/l	277.0 (203.0, 327.0)	253.0 (206.0, 331.5)	310.0 (243.0, 395.0)	0.062
Vitamin D nmol/l	30.5 (22.0, 44.0)	32.0 (23.0, 40.0)	32.5 (22.0, 47.0)	0.73
Ferritin mmol/l	152.1 (49.1, 354.6)	222.6 (78.5, 392.2)	198.2 (118.3, 337.3)	0.52
DDimer mmol/l	0.4 (0.3, 0.7)	0.4 (0.2, 0.6)	0.5 (0.3, 1.0)	0.27
Lactate, mmol/l	1.4 (1.1, 1.8)	1.6 (1.1, 2.1)	1.7 (1.2, 2.0)	0.27

*P-values are based on Kruskal–Wallis test.

Following completion of baseline testing, all patients were followed during their hospital stay until discharge or mortality or for a maximum of 14 days. In cases where the patient remained hospitalized after Day 14, no further data was collected, except for discharge, mortality, and readmission data at Day 30. Patients randomised in each therapeutic group did not receive any antiviral agents other than the therapies allocated to their specific treatment group. Patients in the control group were managed with supportive therapy alone. Patients were assessed routinely for the presence of any significant deterioration. Deterioration criteria increased oxygen requirements to maintain more than 95% saturation, respiratory distress, or worsening chest infiltrates on chest X-ray. Those that deteriorated were treated with steroids (dexamethasone or methylprednisolone) as per Bahrain COVID-19 protocol. Rescue therapy was the administration of Tocilizumab (to be administered as per local tocilizumab protocol).

### Primary outcome measure

The clinical scale at the end of study follow up (day 14 or on discharge/death, whichever is earlier) that was defined as 6 points: death; (5) points: hospitalization plus extracorporeal membrane oxygenation (ECMO) or invasive mechanical ventilation; (4) points: hospitalization plus non-invasive ventilation or high-flow supplemental oxygen; (3) points: hospitalization plus supplemental oxygen (not high-flow or non-invasive ventilation); (2) points: hospitalization with no supplemental oxygen; (1) point: hospital discharge.

### Secondary outcome measures

Secondary outcome measures included viral clearance (defined as a single negative SARS-CoV2 PCR nasopharyngeal result), discharge and length of hospital stay, 30 days readmission rate, 30 days mortality rate, Daily Sequential Organ Failure Assessment (SOFA) score^17^, Daily National Early Warning Score (NEWS) 2 score^18^, the requirement of escalation of respiratory support, clinical improvement defined as patient discharge or a reduction of 2 points on a 6-point disease severity clinical scale, need of ICU Care, adverse events, changes in laboratory measures; C reactive protein, lactate dehydrogenase, ferritin, D-dimer and lactate changes.

### Laboratory measurements

White blood cell count was measured by flow cytometry, lactate dehydrogenase (LDH) was measured using a kinetic method, C-reactive Protein (CRP) and D-dimer measured by an immuno-turbidimetric assay; ferritin was measured by an electrochemiluminescence immunoassay according to the manufacturers’ instructions.

### Statistical analysis

There are currently no robust studies in humans that allow for a definitive power calculation. From preliminary findings by Gautret et al.^19^ and sample size estimation guide for pilot studies reported by Birkett and Day^20^, 50 patients per arm would allow 80% power to detect a difference in the proportion that recovers (based on viral clearance) between two groups, assuming 5% alpha error and 10% attrition rate. The protocol was amended to incorporate clinical scale as the primary outcome since it is a related measure of recovery. Since there are no studies available for this outcome, the previous sample size calculation was considered applicable.

Baseline continuous data are presented as medians and inter-quartile ranges. Intention to treat analysis was used throughout. The proportion of patients who achieved a clinical severity scale of less than 2 and the proportion of patients who achieved viral clearance was compared between the arms using logistic regression. Length of stay was compared between the arms using Kaplan Meier analysis.

For all statistical analyses, a two-tailed P < 0.05 is considered to indicate statistical significance.

Statistical analysis was performed using Stata (StataCorp. 2021. *Stata Statistical Software: Release 17*. College Station, TX: StataCorp LLC.").

## Results

The participant demographic and clinical characteristics in Table 1 and the biochemical characteristics are shown in Table 2. The three groups showed similar baseline epidemiological characteristics. The blood count and biochemical profile were comparable at baseline across the treatment groups, except for urea, lower in the hydroxychloroquine group (p = 0.002) than the other two groups.

### Primary outcome

**Figure 2:**
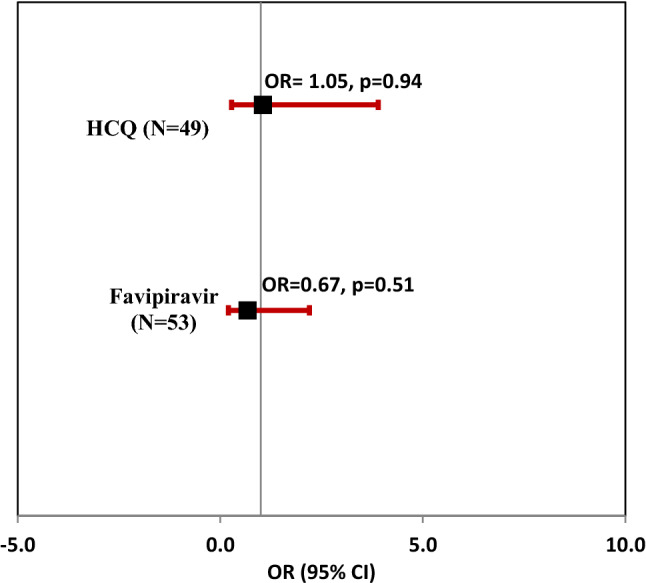
Odds of achieving a clinical scale score < 2 were similar for all the groups at the end of the study period.

The proportion of patients who recovered based on a clinical scale < 2 at the end of study follow-up was similar across the groups (Table 3). The odds of achieving a clinical scale score of less than 2 were similar across all the groups (Fig. 2). Mortality was observed only in one patient in the favipiravir group. The patient was a 77-year-old female with pre-existing diabetes and morbid obesity. Rescue therapy was given to only one patient, a 70-year-old female in the standard care group. No readmissions occurred in any of the groups. ICU support was required only for 8 patients, of which only 1 was in the favipiravir group, 3 were in HCQ, and 4 were given standard care.

**Table 3:** Main outcomes and clinical parameters at the end of the study period.

Parameter	Group			*P*-value
	Favipiravir	Hydroxychloroquine	Standard Care	
N	54	51	52	
Clinical scale < 2, N = 149 **#** [n(%)]	45 (84.9%)	44 (89.8%)	42 (89.4%)	0.702
Viral Clearance, N = 118 **#** [n(%)]	32 (80%)	31 (81.6%)	26 (65%)	0.168
Median length of hospital stay, days [median (IQR)] *	6 (4, 9)	6 (4, 8)	7 (4.5,9)	0.126
SOFA Scale Score, [median (IQR)]	0.0 (0.0, 0.1)	0.0 (0.0, 0.3)	0.0 (0.0, 0.3)	0.42
NEWS2 Scale Score, [median (IQR)]	0.0 (0.0, 1.0)	1.0 (0.0, 2.0)	0.0 (0.0, 1.0)	0.20
FiO2 (fraction of inspired oxygen), [median (IQR)]	0.2 (0.2, 0.2)	0.2 (0.2, 0.2)	0.2 (0.2, 0.2)	0.39
Temperature (C), [median (IQR)]	36.7 (36.6, 36.9)	36.6 (36.5, 36.8)	36.7 (36.6, 36.8)	0.22
Diastolic BP (mmHg), [median (IQR)]	72 (69, 80)	68 (63, 74)	76 (68, 82)	**0.005**
Systolic BP (mmHg), [median (IQR)]	120 (110, 129)	115 (107, 123)	125 (113, 134)	**0.005**
Respiratory Rate (breaths/min), [median (IQR)]	20.0 (18.0, 20.0)	18.0 (18.0, 20.0)	18.0 (18.0, 20.0)	0.81
Heart Rate (beats/min), [median (IQR)]	78.0 (68.0, 84.0)	75.0 (70.0, 82.0)	78.0 (72.0, 84.0)	0.67
Oxygen Saturation (mm Hg), [median (IQR)]	98.0 (97.0, 99.0)	98.0 (97.0, 99.0)	97.0 (97.0, 99.0)	0.12

^#^*P* values for viral clearance and clinical scale are based on chi-square test.

*P-value for a median length of stay is based on the log-rank test.

*P* values for the rest of the variables are based on Kruskal–Wallis test.

SOFA -Daily Sequential Organ Failure Assessment = SOFA score^17^.

### Secondary outcomes

**Figure 3:**
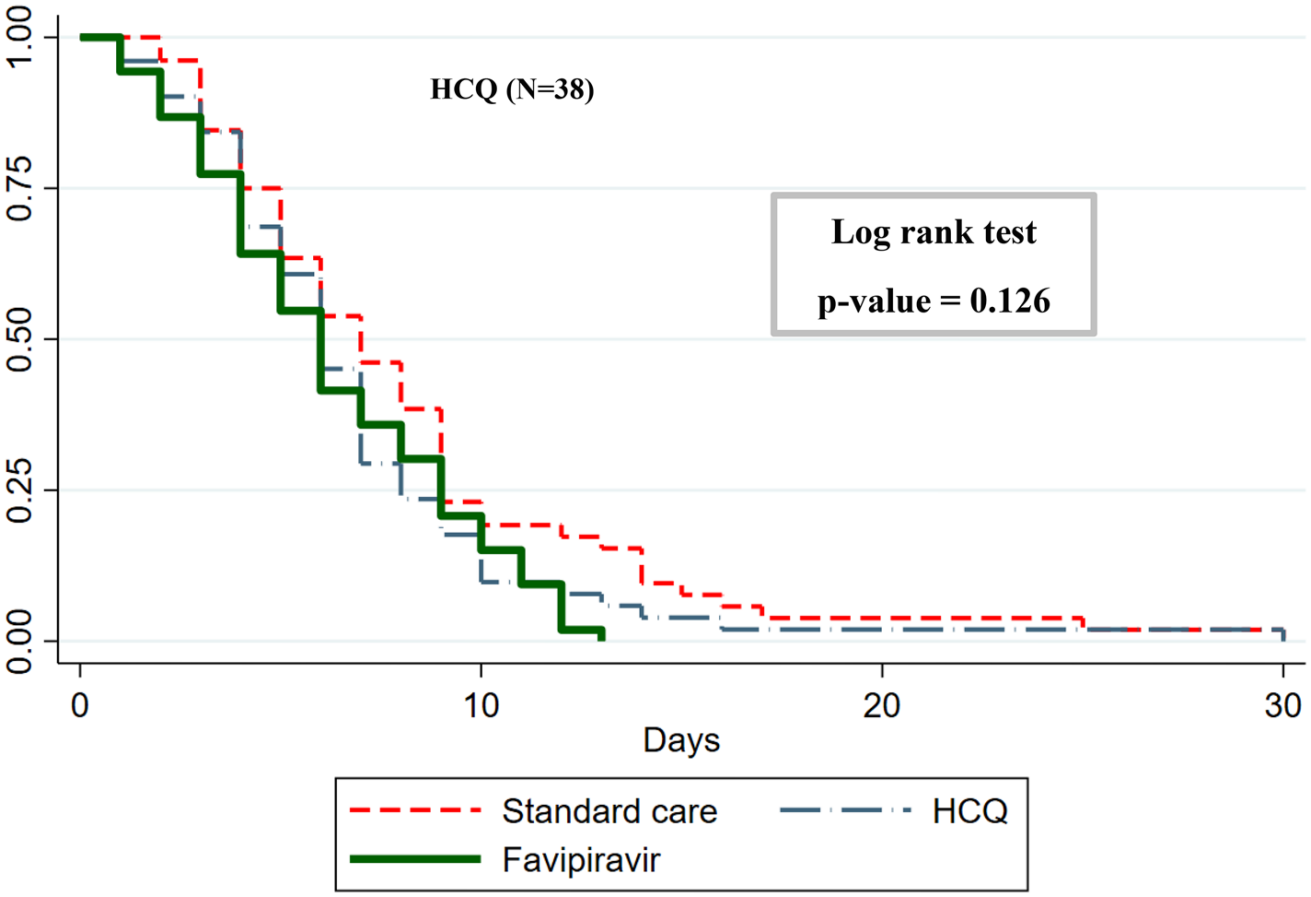
The median length of hospital stay (Table 3) was not significantly higher (6 days) for both HCQ and Favipiravir groups, compared to patients on standard care (7 days).

The proportion of patients who achieved viral clearance was comparable across all the groups (Table 3). There was no difference between median NEWS2 or SOFA scores across the groups either (Table 3). The only other secondary outcomes that differed between groups were diastolic and systolic blood pressure lower in the hydroxychloroquine group (< 0.005), as shown in Table 3. The median length of hospital stay (Fig. 3, Table 3) was marginally lower (6 days) for both HCQ and favipiravir groups, compared to patients on standard care (7 days).

The biochemical profile of the subjects remained similar across the treatment groups at the end of the study period, except for the platelet counts and uric acid, which were higher in the favipiravir group (p < 0.03 and p < 0.004, respectively) as shown in Table 4.

**Table 4:** Blood Count and Biochemical Profile of the participants at discharge [median (IQR)].

Factor	Favipiravir	Hydroxychloroquine	Standard Care	*P*-value
N	54	51	52	
Sodium mmolL, median (IQR)	138.0 (137.0, 140.5)	138.7 (138.0, 141.0)	139.0 (137.0, 140.3)	0.33
Potassium mmol, median (IQR)	4.3 (4.1, 4.6)	4.3 (4.1, 4.7)	4.3 (4.1, 4.7)	0.69
Urea mmolL, median (IQR)	4.1 (3.6, 5.4)	4.1 (3.5, 5.3)	4.6 (4.0, 5.7)	0.10
Creatinine umolL, median (IQR)	58.0 (50.1, 66.2)	56.8 (49.0, 68.8)	56.7 (49.0, 65.4)	0.98
Hemoglobin mg/dl, median (IQR)	13.0 (11.5, 14.1)	13.7 (12.0, 14.9)	13.4 (12.0, 14.6)	0.38
White blood count median (IQR)	5.6 (4.1, 6.7)	5.4 (4.4, 7.1)	5.3 (4.0, 7.2)	0.85
Neutrophil Count, median (IQR)	3.0 (2.0, 4.0)	3.3 (2.0, 4.0)	3.0 (2.0, 4.0)	0.98
Lymphocyte Count, median (IQR)	1.5 (1.1, 2.0)	1.5 (1.2, 2.0)	1.5 (1.1, 1.8)	0.69
Platelet Count, median (IQR)	258 (209, 318)	217 (180, 284)	243 (176, 272)	**0.029**
Lactate dehydrogenase mmolL, median (IQR)	212 (196, 256)	197 (163, 265)	240 (175, 304)	0.19
C-reactive protein mmolL, median (IQR)	16.6 (4.1, 47.5)	9.5 (3.9, 25.5)	16.7 (5.8, 39.7)	0.26
Uric Acid mmolL, median (IQR)	313 (256, 370)	247 (185, 315)	265 (198, 337)	**0.004**
Ferritin mmolL, median (IQR)	128 (66, 383)	250 (76, 460)	241 (100, 590)	0.34
DDimer mmolL, median (IQR)	0.4 (0.3, 0.8)	0.5 (0.3, 0.9)	0.5 (0.3, 0.9)	0.63
Lactate mmolL, median (IQR)	1.3 (1.1, 1.8)	1.7 (1.3, 2.0)	1.7 (1.3, 2.3)	0.11
Bilirubin umol/l, median (IQR)	6.2 (4.9, 9.5)	6.8 (5.5, 9.9)	6.8 (5.2, 9.8)	0.36

*Significance values are based on Kruskal–Wallis test.

### Adverse reactions

Favipiravir: one patient with deranged liver function tests (elevated ALT three times the upper limit of normal) and one patient with severe headache were documented and resulted in stopping favipiravir. Hydroxychloroquine: two patients with deranged liver function tests led to stopping therapy; QT prolongation (ranging from 493 to 446) was seen in three subjects receiving HCQ; two patients continued taking the medication, while the third developed deranged liver function tests first and then prolonged QT segment, resulting in the hydroxychloroquine being stopped. All adverse events were resolved.

## Discussion

In February 2020, favipiravir was used to treat COVID-19 disease in China and was declared effective in treatment^14^, and a report published comparing favipiravir with lopinavir /ritonavir suggested that favipiravir was superior for the prevention of disease progression and viral clearance^15^. Each group was well matched in this pilot study, as shown by the demographic, clinical, and biochemical baseline parameters. There were no differences in the primary outcome clinical measures seen for either favipiravir or hydroxychloroquine therapy in this COVID-19 patient population in comparison to standard supportive care. Hospital stay was marginally less at six days with intervention versus seven days for standard therapy, there were no differences in progression of disease with ventilation or death, and the measures of improvement in clinical scale, NEWS2 scale, and the SOFA scale score did not differ. The primary outcome of this pilot study was to determine a difference in clinical severity scale across three groups, and the results suggest that a study with 80% power would require 270 subjects in total for three arms.

Subjects in the favipiravir, as well as the hydroxychloroquine group, were more than twice as likely to test negative for COVID-19 at the end of the study period compared to participants in the standard care group as indicated by the odds ratios (supplementary Fig. S1); however, this difference was not statistically significant. Udwadia et al.^21^ performed a randomized, open-label, parallel-arm, multicenter, phase 3 trial to test favipiravir’s ability to reduce viral load of SARS-CoV-2 and secondarily to test its ability to improve clinical parameters, the results of which showed that there was some clinical benefit of favipiravir, but no significant effect on viral load. The study here and the Udwadia study concur that there is no significant effect provided by favipiravir treatment in SARS-CoV-2 disease and they both contradict the efficacy shown by Cai et al.^15^ where shorter viral clearance and improved computed tomography changes were seen with favipiravir. Notably, because there were observed non-statistically significant positive trends in the study here and Udwadia et al., it is possible that that the small size of both studies may have compromised statistical power resulting in a lack of significant positive effect of favipiravir in COVID-19. These results were in accord with that of a recent meta-analysis on favipiravir versus control showing a non significant decrease in viral load with no significant effect on symptoms in those with mild or moderate COVID-19^22^. Overall, this would suggest that favipiravir has no place in the therapy of mild to moderate COVID-19 disease, though whether it may be of benefit in severe disease is unclear^22^.

Of note, only the systolic and diastolic blood pressure measurements were lower with hydroxychloroquine therapy that may potentially be of benefit as COVID-19 patients’ respiratory failure is associated with increased systemic blood pressure^23^. Similarly, there was no difference in the biochemical parameters between the three subject groups, except for the platelet count and uric acid, higher in the favipiravir group. An increased platelet count has been reported for favipiravir therapy with the suppression of the erythrocyte series, and a fall in hemoglobin that was not seen to be statistically significant here^24^, and the elevation in uric acid by favipiravir has been described^25^; gout is a contraindication to its use.

Favipiravir was found to be safe in this patient population that had been screened for potential contraindications of gout, neutropenia/blood count abnormalities, elevated liver function tests, glycosuria, hyperkalemia. Favipiravir is a known teratogen. The oral tablet cost is relatively modest^12^, but without any overt clinical benefits, then monotherapy would not appear to have a health economic benefit.

The COVID-19 patients in this study were mainly mild, though symptomatic, and relatively few deteriorated; therefore, it is unclear whether favipiravir therapy in a more severe COVID-19 cohort would have greater benefits, though the results shown here would suggest that this would be unlikely. What cannot be answered by this trial is whether favipiravir in other combination therapy would have an additive therapeutic effect that has been suggested in other studies where favipiravir was combined with steroids^26^.

Hydroxychloroquine has shown in vitro activity against SAR-CoV-2^27^, with an initial trial that showed improvement in these patients^5^. A subsequent study showed increased viral clearance that would be in accord with the finding here^19^, but others showed no clinical difference^7^ in mild to moderate disease, and a large observational study showed no differences in intubation or death^6^, in accord with the no difference in clinical findings here compared to standard treatment. The general consensus from several studies testing hydroxychloroquine efficacy, did not decrease mortality nor increase viral clearance and resulted in more adverse events Geleris et al.^28^ Ghazy et al.^29^: recruitment of this study reported here using a randomized design began in August 2020 when hydroxychloroquine was still being studied and prior to the evidence that showed its lack of efficacy, and hence its inclusion as an arm of this study protocol.

### Strength and limitations

The main strength of the study confirmed the safety of using favipravir in treating mild COVID-19 patients as well as increasing viral clearance in accord with the results of Udwadia et al.^21^, though noting the increase in the uric acid levels in favipravir treated patients. This study was conducted at a time when data was lacking and it provides further support to the now existing research that favipiravir may not be efficacious in mild and moderate COVID-19 disease. The limitations of this study were the relatively small number of subjects, but that was in accord with this being a pilot study. Including more subjects over 65 years and or including those with more severe COVID-19 disease may have led to a greater difference in clinical outcome as the increased viral load is associated with increased disease severity^30^ and viral clearance is associated with mortality in this age group^31^. Additionally, the nationalities and ethnicity of the patients were not taken into account in this study that would need to be addressed in an appropriately powered study.

### Future research recommendations

As noted above this study together with the results of the Udwadia et al.^21^ study and recent meta-analysis^22^ would suggest that if favipiravir alone has a role in COVID-19 disease then it needs to be determined in severe COVID-19 disease with a large randomized control trial. Furthermore, increasing the heterogenicity of the research subjects while noting the demographic differences and including more subjects over the age of 65 years would be important. The evaluation of favipiravir in combination with newer antivirals such as molnupiravir to test the potentiation efficacy of such a combination may be of value. Finally, as mentioned the limitations section including more subjects over 65 years and or including those with more severe COVID-19 disease may lead to a greater difference in clinical outcome.

## Conclusion

In conclusion, there were no significant differences in the primary or secondary outcome measures between favipiravir, hydroxychloroquine, and standard therapy in patients with mild to moderate COVID-19 disease. However, favipiravir therapy appeared safe, and while there was a trend to increased viral clearance, there was no superior therapeutic utility.

## Supplementary Information

Supplementary Information.

## Data Availability

Most of the data generated and analyzed during this study is included in this published article. However, some data is not publicly available to maintain the confidentiality of the participants but can be provided from the corresponding author upon reasonable request.
