# Author Correction: Randomized controlled trial of favipiravir, hydroxychloroquine, and standard care in patients with mild/moderate COVID-19 disease

**DOI:** 10.1038/s41598-022-20899-w

**Published:** 2022-09-26

**Authors:** Manaf AlQahtani, Nitya Kumar, Dhuha Aljawder, Abdulkarim Abdulrahman, Mohammed Wael Mohamed, Fatema Alnashaba, Mohammed Abu Fayyad, Faisal Alshaikh, Fatima Alsahaf, Sawsan Saeed, Amal Almahroos, Zainab Abdulrahim, Sameer Otoom, Stephen L. Atkin

**Affiliations:** 1 National Task Force for Combating the Corona Virus (COVID-19), Riffa, Bahrain; 2 Bahrain Defence Force Hospital, Riffa, Bahrain; 3 Royal College of Surgeons in Ireland, Riffa, Bahrain; 4 Salmaniya Medical Complex, Manama, Bahrain; 5 Sheikh Mohammed Bin Khalifa Cardiac Centre, Riffa, Bahrain; 6 King Hamad University Hospital, Muharraq, Bahrain

Correction to: *Scientific Reports* 10.1038/s41598-022-08794-w, published online 23 March 2022

Mohammed Wael Mohamed was omitted from the author list in the original version of this Article.

The Author Contributions section now reads:

“N.K.: data analysis, interpretation and preparation of the manuscript; D.A., A.A., F.A., M.A.F., F.A., F.A., S.S., A.A., Z.A., S.O. S.A. & M.M.: conception and design, data collection, manuscript review; S.S., N.K. & S.L.A.: writing and manuscript review; M.A.Q.: conception and design of the study and manuscript review.”

The original Article has been corrected.

