# Supplementary material for: Randomized controlled trial of favipiravir, hydroxychloroquine, and standard care in patients with mild/moderate COVID-19 disease
Source: Sci Rep. 2022 Mar 23;12:4925. doi: 10.1038/s41598-022-08794-w (PMC8943168; doi:10.1038/s41598-022-08794-w)

Supplementary Material

Supplementary Figure S1: **Odds of Viral Clearance (Secondary Outcome) in Treatment Arms Compared to Standard Care**

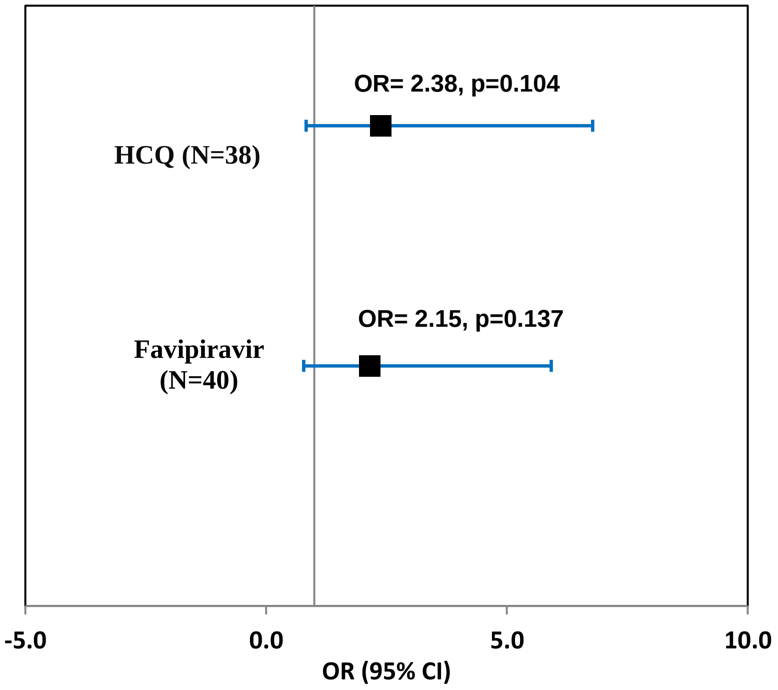

Supplement: Supplementary file 1 — Supplementary Information. [file 41598_2022_8794_MOESM1_ESM.pdf]
